# Resemblance of the human liver sinusoid in a fluidic device with biomedical and pharmaceutical applications

**DOI:** 10.1002/bit.26776

**Published:** 2018-07-13

**Authors:** Martí Ortega‐Ribera, Anabel Fernández‐Iglesias, Xavi Illa, Ana Moya, Víctor Molina, Raquel Maeso‐Díaz, Constantino Fondevila, Carmen Peralta, Jaume Bosch, Rosa Villa, Jordi Gracia‐Sancho

**Affiliations:** ^1^ Liver Vascular Biology Research Group, Barcelona Hepatic Hemodynamic Laboratory IDIBAPS Biomedical Research Institute Barcelona Spain; ^2^ Biomedical Applications Group (GAB) Institut de Microelectrònica de Barcelona, IMB‐CNM (CSIC), Esfera UAB Bellaterra Spain; ^3^ Biomedical Research Networking Center in Hepatic and Digestive Diseases (CIBEREHD) Madrid Spain; ^4^ Biomedical Research Networking Center in Bioengineering Biomaterials and Nanomedicine (CIBERBBN) Madrid Spain; ^5^ Liver Surgery and Transplantation Unit, IDIBAPS Hospital Clínic de Barcelona Barcelona Spain; ^6^ Protective Strategies Against Hepatic Ischemia‐Reperfusion Group, IDIBAPS Barcelona Spain; ^7^ Hepatology, Department of Biomedical Research, Inselspital Bern University Bern Switzerland

**Keywords:** hepatocyte, liver‐on‐a‐chip, liver sinusoidal endothelial cells, LSEC, sinusoid

## Abstract

Maintenance of the complex phenotype of primary hepatocytes in vitro represents a limitation for developing liver support systems and reliable tools for biomedical research and drug screening. We herein aimed at developing a biosystem able to preserve human and rodent hepatocytes phenotype in vitro based on the main characteristics of the liver sinusoid: unique cellular architecture, endothelial biodynamic stimulation, and parenchymal zonation. Primary hepatocytes and liver sinusoidal endothelial cells (LSEC) were isolated from control and cirrhotic human or control rat livers and cultured in conventional in vitro platforms or within our liver‐resembling device. Hepatocytes phenotype, function, and response to hepatotoxic drugs were analyzed. Results evidenced that mimicking the in vivo sinusoidal environment within our biosystem, primary human and rat hepatocytes cocultured with functional LSEC maintained morphology and showed high albumin and urea production, enhanced cytochrome P450 family 3 subfamily A member 4 (CYP3A4) activity, and maintained expression of hepatocyte nuclear factor 4 alpha (*hnf4α*) and transporters, showing delayed hepatocyte dedifferentiation. In addition, differentiated hepatocytes cultured within this liver‐resembling device responded to acute treatment with known hepatotoxic drugs significantly different from those seen in conventional culture platforms. In conclusion, this study describes a new bioengineered device that mimics the human sinusoid in vitro, representing a novel method to study liver diseases and toxicology.

## 1 INTRODUCTION

Primary hepatocytes are highly specialized cells used as the main tool for assessing hepatotoxicity, cellular transplantation, biomedical research, and as an essential component of active bioartificial devices to support liver function (Baccarani et al., 2004; Godoy et al., 2013; Nicolas et al., 2017). Nevertheless, specific functions and differentiated phenotype are progressively lost when hepatocytes are cultured in vitro, leading to loss of enzymatic activity and detoxification capacity, changes in cell morphology and function, and deregulation of transporters expression (Elaut et al., 2006; Rowe et al., 2010). Several approaches have been proposed to overcome/delay this dedifferentiation process, including sandwich cultures, spheroid systems, or the development of sinusoidal‐mimicking devices known as liver‐on‐a‐chip (Fraczek, Bolleyn, Vanhaecke, Rogiers, & Vinken, 2013; Lauschke, Hendriks, Bell, Andersson, & Ingelman‐Sundberg, 2016).

Liver‐on‐a‐chip are usually low‐volume miniaturized devices that enable the culture of hepatic cells in different configurations both under flow or static conditions. In a healthy liver, hepatocyte functions are partially maintained by microenvironmental signaling from neighboring cells; for this reason, hepatocytes within these liver‐resembling devices are often studied in coculture with nonparenchymal cells. Liver sinusoidal endothelial cells (LSEC), hepatic macrophages, and hepatic stellate cells constitute the major populations of nonparenchymal cells in the liver (Arias et al., 2009; Wisse et al., 1996). They play central roles both in liver physiology and pathology, and therefore cannot be ignored to generate reliable coculture systems (Marrone, Shah, & Gracia‐Sancho, 2016; Usta et al., 2015), and to guarantee a greater translational capability in studies using human liver cells.

Considering the above‐mentioned background, the design, development, and future applicability of a liver‐on‐a‐chip device requires accurate selection of the hepatic cell type to be cultured, as well as the internal and external environmental stimuli that will modulate the phenotype of hosted cells (Fraczek et al., 2013). Although several authors used immortalized human cell lines to substitute fresh hepatocytes within their microfluidic devices (Bavli et al., 2016; Ma et al., 2016; Rennert et al., 2015) these lack a significant part of liver‐specific functions (Kanebratt & Andersson, 2008; Wilkening, Stahl, & Bader, 2003). Regarding the microenvironment modulating hepatocytes phenotype, in nature parenchymal cells are partly maintained through paracrine communication from LSEC. Indeed, the key role of LSEC in the liver has been patent not only for being the first cells sensing liver injury (Hide et al., 2016; McCuskey, 2006) but also for maintaining and enhancing hepatocytes phenotype (Bhatia, Balis, Yarmush, & Toner, 1999; Kasuya, Sudo, Mitaka, Ikeda, & Tanishita, 2011; Liu, Li, Yan, Wei, & Li, 2014; Marrone et al., 2016). In the specific field of liver‐on‐a‐chip, LSEC have been questionably replaced by general endothelial cells, such as human aortic endothelial cells (HAEC) (Ma et al., 2016), human umbilical vein endothelial cells (HUVEC) (H. Lee & Cho, 2016; J. W. Lee et al., 2016; Rennert et al., 2015), stable human endothelial cell line (EA.hy926) (Prodanov et al., 2016) or BAEC (Kang et al., 2015) among others. However, primary LSEC are rarely found in this context, especially when using human liver cells.

We hypothesized that maintaining a physiological sinusoid‐like environment allowing the paracrine communication between hepatocytes and functional LSEC would provide a suitable milieu for maintaining the phenotype and function of these cells, delaying hepatocyte dedifferentiation, and being more sensitive in predicting hepatotoxicity than conventional two‐dimensional in vitro cultures. To test this hypothesis, and mainly focusing on its translational applicability, the primary aim of our study was to cautiously characterize the phenotype and function of primary human hepatocytes cocultured with primary functional human LSEC within a fluidic device that mimics the hepatic sinusoid (Illa et al., 2014) and compare with conventional configurations. In addition, and as a secondary aim, we studied this liver‐on‐a‐chip as a potential tool for preclinical research on the fields of chronic liver disease and hepatotoxicity. Supplementary experiments using primary rat cells were performed to endorse the model in a non‐human experimental scenario.

## 2 MATERIALS AND METHODS

### 2.1 Isolation of human and rat hepatocytes and LSEC

Human cells were isolated from remnant tissue approximately weighing 20 g obtained after human partial hepatectomy to excise tumor metastasis from colon carcinoma (for healthy cells; note that obtained peritumoral tissue was confirmed as “normal” by anatomical pathologists) and from the discarded tissue after liver transplantation (chronic ethanol etiology, for cirrhotic cells). Ethics Committee of the Hospital Clínic de Barcelona approved the experimental protocol (HCB/2015/0624), and in all cases, patients received and agreed to an informed consent.

Rodent cells were isolated from male Wistar Han rats (Charles River Laboratories Barcelona, Spain) weighing 300–350 g kept at the University of Barcelona Faculty of Medicine facilities with controlled temperature (19.7 ± 2°C), humidity (52 ± 5%) and light/dark cycle (12 hr each). Animals were fed ad libitum with water and standard rodent food pellets. All experiments were approved by the Laboratory Animal Care and Use Committee of the University of Barcelona and were conducted in accordance with the European Community guidelines for the protection of animals used for experimental and other scientific purposes (European Economic Community Directive 86/609).

Hepatocytes and LSEC were isolated using standardized protocols (Gracia‐Sancho et al., 2007; Oie, Snapkov, Elvevold, Sveinbjornsson, & Smedsrod, 2016) and cultured as detailed in Supporting Information Materials. Highly pure and viable cells were used. Cell density under each individual experimental condition was 10^6^ hepatocytes and 2.5·10^5^ LSEC.

### 2.2 Liver‐on‐a‐chip technology and culture of primary cells

**Figure 1:**
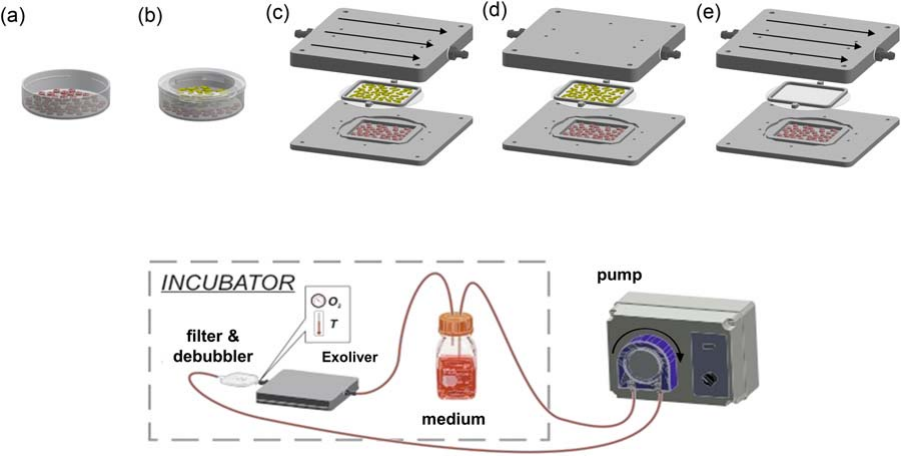
Experimental conditions analyzed. Top, in vitro conventional culture methods: (a) monoculture in 35 mm petri dish and (b) coculture in transwell. Exoliver conditions: (c) dynamic coculture (optimal condition), (d) static coculture, and (e) dynamic monoculture. Hepatocytes represented in red and liver sinusoidal endothelial cells (LSEC) in yellow. Bottom, Exoliver design and circuit components [Color figure can be viewed at wileyonlinelibrary.com]

Our team has recently developed a fluidic device whose detailed fabrication and features were previously described in Illa et al. (2014) and is herein termed Exoliver. Briefly, it consists of a sinusoidal‐mimicking layered structure that allows coculture of different cell types and fluidic stimulation of the top layer of the device. LSEC were grown in the upper area on a hydrophilic polytetrafluoroethylene microporous membrane with homogeneous and continuous shear stress stimulation, whereas hepatocytes were plated in the lower poly (methyl methacrylate) area of the device. Dynamic Exoliver configurations started with a shear stress stimulus of 0.1 dyn/cm^2^ that was gradually increased during the first 2 hr of culture until reaching 1.15 dyn/cm^2^ (1.5 ml/min), with a total amount of 43 ml unidirectional recirculating culture media. Exoliver, reservoir, filters, and most of the tubing were placed inside an incubator to maintain physiological conditions (37°C, 5% CO_2_). Five different experimental configurations were considered for this study (Figure 1).

The day after the isolation, hepatocytes and LSEC were rinsed twice with the Dulbecco phosphate‐buffered saline (02‐023‐1A; Reactiva), and media was changed to Dulbecco modified Eagle medium (DMEMF12; 11320074; Gibco) supplemented with 2.97% dextran (31392; Sigma, Darmstadt, Germany) to simulate blood viscosity, 2% fetal bovine serum (04‐001‐1A; Reactiva), 1% penicillin plus 1% streptomycin (03‐331‐1C; Reactiva), 1% endothelial cell growth supplement (BT‐203; Biomedical Technologies), 1% heparin (H3393; Sigma), 1% l‐glutamine (25030‐024; Gibco), 1% amphotericin B (03‐029‐1C; Reactiva), 1 nM dexamethasone (D4902; Sigma), 10 ng/ml Epidermal Growth Factor (E4127; Sigma), 1.5 nM glucagon (16941‐32‐4, Novo Nordisk), 15 nM hydrocortisone (H0888, Sigma), and 1 µM insulin (Humulin S, Lilly S.A.).

Then, transwells and bioreactors were assembled and perfusion of the dynamic conditions started. Human and rat cultures were maintained for 3 or 7 days, and then disassembling of the bioreactor was performed to separately analyze both cell types. Cell supernatant analysis, quantitative polymerase chain reaction, and CYP3A4 assay were performed under all experimental conditions mentioned above, as described in Supporting Information Methods.

Once concluded that there were no significant differences in the studied markers between conventional mono‐ and coculture configurations, we decided to eliminate the conventional coculture condition in the 7‐day human experiments to maximize cell seeding under the other conditions obtained from the scarce liver tissue available after surgery.

### 2.3 Statistics and data analyses

Statistical analysis was performed with SPSS Statistics19 software for Windows. Results were expressed as mean ± standard error of mean. To assess differences between groups, we performed one‐way analysis of variance with least significant difference (LSD) post‐hoc tests when variables were parametric and Mann–Whitney test for nonparametric variables. Differences between groups were considered as significant when *p* value ≤ 0.05. Each experiment was performed in duplicate from at least *n* = 3 independent isolations.

## 3 RESULTS

### 3.1 Exoliver maintains human hepatocyte phenotype and function

Maintenance of human healthy hepatocytes phenotype was assessed under five experimental conditions (Figure 1, top): hepatocytes cultured in two conventional configurations (monoculture and coculture with LSEC) and within Exoliver in three different configurations: coculture with LSEC stimulated with continuous and homogenous shear stress (optimal condition), coculture without shear stress (which leads to LSEC dysfunction Supporting Information Figure 1) and hepatocytes monoculture with indirect flow stimulus (without paracrine interactions from LSEC).

**Figure 2:**
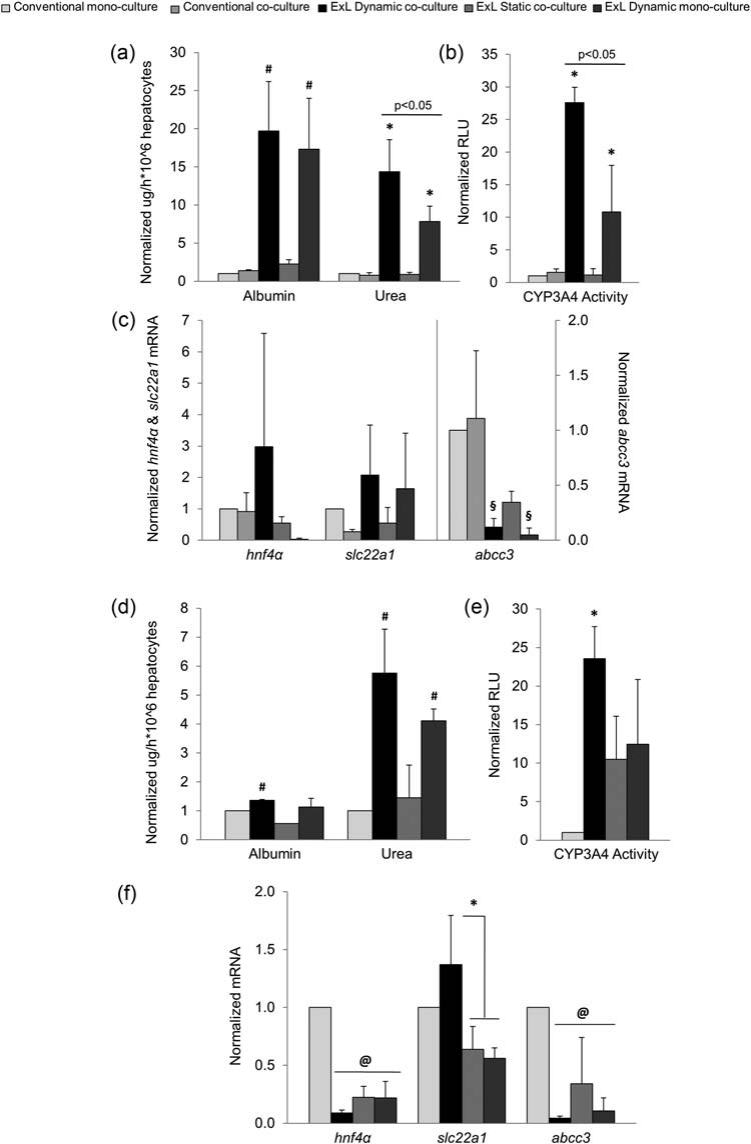
Evaluation of healthy primary human hepatocytes after 3 days (a–c) or 7 days (d–f) of culture under the experimental conditions described in Figure 1. Synthetic capacity of hepatocytes was measured as albumin and urea secretion, phase I enzymes detoxification capacity as cytochrome P450 family 3 subfamily A member 4 (CYP3A4) activity and cell phenotype markers as gene expression of the transcription factor hepatocyte nuclear factor 4 alpha (*hnf4α*), solute carrier family 22 member 1 (*slc22a1*), and ATP‐binding cassette subfamily C member 3 (*abcc3*) transporters. Data derive from *n* = 4 independent experiments were normalized to conventional monoculture condition (fold change of 1) and expressed as mean ± standard error of the mean. *p* value < 0.05 versus all conditions (*), all static conditions (^#^), both conventional conditions (^§^), or conventional monoculture condition (^@^). ExL: Exoliver

Synthetic capacity of hepatocytes was evaluated as active albumin and urea production and release to the culture media. Human primary hepatocytes cultured under the Exoliver dynamic coculture condition showed higher albumin synthesis when compared with all static conditions after 3 days (Figure 2a) and to a lesser extent after 7 days (Figure 2d) of culture. Hepatocytes cultured using Exoliver dynamic monoculture configuration produced higher albumin than static configurations after 3 days, but this was no longer seen after 7 days of culture. At 3 days of culture, urea production was highly increased in dynamic coculture configurations and partially maintained by dynamic monoculture condition (Figure 2a). At 7 days of culture (Figure 2d), both dynamic conditions showed increased urea production compared with all static configurations.

Primary human hepatocytes in coculture with shear stress‐stimulated LSEC inside Exoliver showed superior cytochrome P450 family 3 subfamily A member 4 (CYP3A4) activity compared with all culture conditions both after 3 and 7 days of culture (Figure 2b,e, respectively). Although CYP3A4 activity in the dynamic monoculture configuration was partially increased after 3 days of culture, this maintenance was no longer significantly different after 7 days of culture, reinforcing the concept of maintenance of hepatocyte function through paracrine interactions from functional LSEC in the dynamic coculture condition.

Hepatocytes phenotype was further assessed by means of expression of the master regulator hepatocyte nuclear factor 4 alpha (*hnf4α*), and the transporters ATP‐binding cassette subfamily C member 3 (*abcc3)* and solute carrier family 22 member 1 (*slc22a1)*. *hnf4α* messenger RNA (mRNA) expression was increased under Exoliver dynamic coculture condition after 3 days (Figure 2c) although prevention of its downregulation was not reached after 7 days of culture (Figure 2f). Results derived from *slc22a1* analyses showed no significant differences in any analyzed group at 3 days of study but exhibited higher expression of this marker under Exoliver dynamic coculture condition compared with suboptimal Exoliver configurations after 7 days of culture. *Abcc3* mRNA upregulation was prevented under all dynamic Exoliver conditions after 3 and 7 days of culture compared with conventional culture configurations.

Conventional configurations showed no significant differences in any of the studied parameters neither after 3 days nor 7 days of culture.

Maintenance of hepatocytes phenotype using this liver‐on‐a‐chip device was confirmed in a second species. Supporting Information Figure 2 shows all data regarding coculture of rat primary hepatocytes and LSEC.

### 3.2 Exoliver prevents hepatocytes morphology deterioration

**Figure 3:**
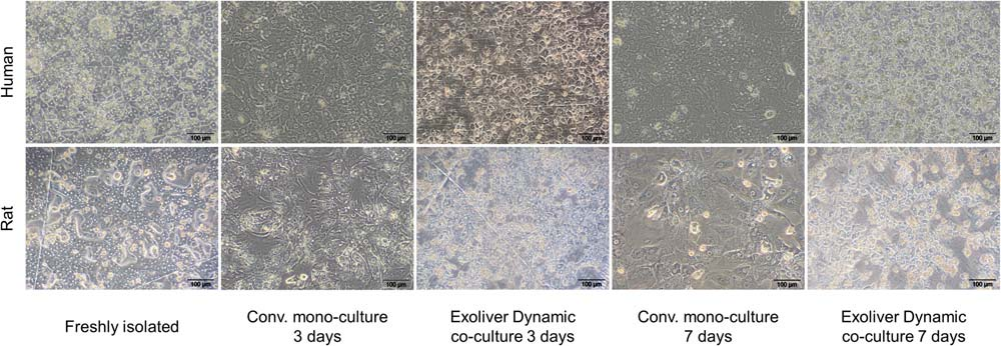
Primary healthy human and rat hepatocytes morphology after culture for 3 or 7 days in conventional monoculture or Exoliver optimal configuration. Images were taken at 10× magnification [Color figure can be viewed at wileyonlinelibrary.com]

Primary human or rat hepatocytes cultured in the previously described conditions exhibited different morphology. The characteristic polygonal shape and angular edges from freshly isolated hepatocytes were gradually lost upon culture in conventional platforms. As shown in Figure 3, hepatocytes became flattened with diffuse separation between cells (Day 3), further acquiring myofibroblast‐like morphology, finally leading to cell aggregation in clusters (Day 7). Prevention of the in vitro dedifferentiation process in the optimal Exoliver configuration was associated with maintenance of hepatocyte polygonal shape both after 3 and 7 days of culture. Suboptimal Exoliver configurations did not maintain hepatocyte morphology (Supporting Information Figure 3). Considering all the collected data, translational experiments of the device were performed using the optimal configuration of the device and compared with conventional cell culture method.

### 3.3 Exoliver as a tool to study chronic liver disease

**Figure 4:**
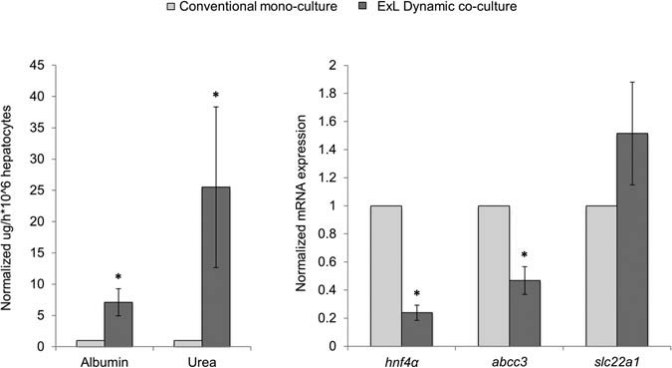
Assessment of cirrhotic primary human hepatocytes after 3 days of culture in conventional monoculture or dynamic coculture using Exoliver. Synthetic capacity of hepatocytes was measured as albumin and urea secretion and cell phenotype markers as gene expression of the transcription factor *hnf4α*, *slc22a1*, and *abcc3*. Data derive from *n* = 4 independent experiments were normalized to conventional monoculture condition (fold change of 1) and expressed as mean ± standard error of the mean. *p value < 0.05 versus conventional culture. ExL: Exoliver

Primary hepatocytes isolated from human cirrhotic livers and cultured in the optimal Exoliver configuration (dynamic coculture with functional LSEC) exhibited significantly better‐preserved phenotype in comparison with cells in monoculture using two‐dimensional conventional methods (Figure 4). Indeed, albumin and urea production and secretion to the culture media was significantly higher in Exoliver‐cultured hepatocytes. Moreover, lower mRNA expression of the transporter *abcc3* and higher mRNA expression of the transporter *slc22a1* were found in hepatocytes cultured using the device, suggesting an overall maintenance of hepatocyte phenotype.

### 3.4 Exoliver as a tool to study hepatotoxicity

Human hepatocytes toxicity response was assessed using acute overdose of the anti‐diabetic drug troglitazone, the catechol‐O‐inhibitor for Parkinson’s disease tolcapone, the nonsteroideal anti‐inflammatory drug diclofenac, and the widely prescribed anti‐pyretic and analgesic drug acetaminophen.

**Figure 5:**
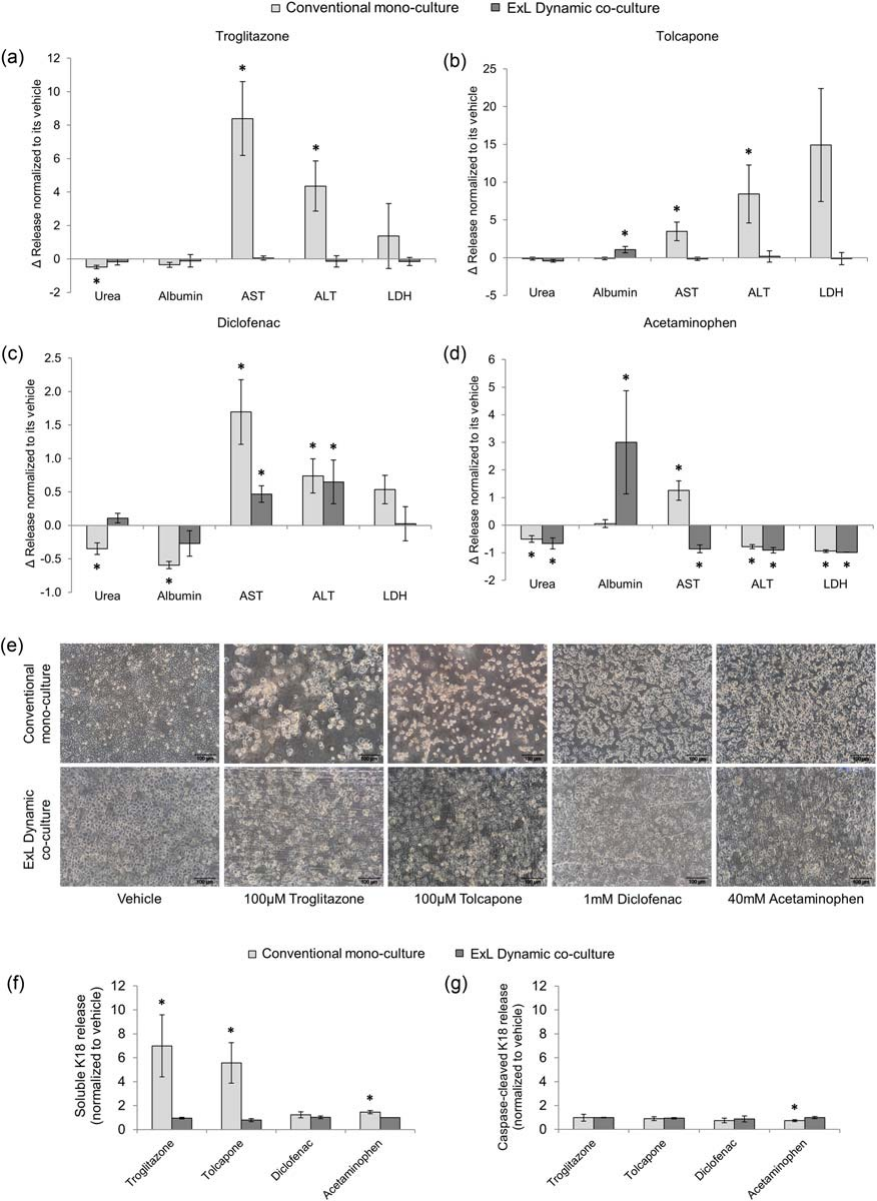
Exoliver‐cultured hepatocytes response to acute drug‐induced injury. Hepatocytes viability was assessed as urea and albumin synthesis and transaminases and LDH release to the culture media. Healthy human hepatocytes were cultured in the optimal Exoliver configuration (with LSEC) or in conventional monoculture. After 24 hr of culture, hepatocytes received acute toxic insult and were cultured for additional 24 hr with 100 μM troglitazone (a), 100 μM tolcapone (b), 1 mM diclofenac (c), or 40 mM acetaminophen (d). Cell morphology (e) and release of cell death markers (soluble keratin 18 and caspase‐cleaved keratin 18) (f,g) were analyzed. Images were taken at 10x magnification. Data derived from *n* = 4 independent experiments were normalized to vehicle concentration (fold change of 1) and expressed as mean ± standard error of the mean. *p value < 0.05 versus its corresponding vehicle. ALT: alanine aminotransferase; AST: aspartate aminotransferase; ExL: Exoliver; K18: keratin 18; LDH: lactate dehydrogenase; LSEC: liver sinusoidal endothelial cells [Color figure can be viewed at wileyonlinelibrary.com]

Hepatotoxic effect of troglitazone (Figure 5a) was demonstrated in hepatocytes cultured in the conventional two‐dimensional configuration; however, it showed no toxic effect on hepatocytes cultured using Exoliver, as demonstrated by the vehicle‐comparable production of the five parameters analyzed. Tolcapone‐derived hepatotoxicity in two‐dimensional cultured hepatocytes was shown as high transaminases release to the culture media (Figure 5b) although no significant effect was detected in albumin and urea production in response to acute treatment with this drug. Hepatotoxicity derived from tolcapone treatment was not seen in Exoliver‐cultured hepatocytes, showing vehicle‐production urea and transaminases and a significant increase in albumin synthesis.

High doses of diclofenac showed toxic effects on hepatocytes (Figure 5 **c**) in conventional cultures as well as in Exoliver‐cultured hepatocytes. Although no significant changes were observed in active urea and albumin synthesis, transaminases were significantly increased in response to acute treatment with this drug.

Results obtained from hepatocytes treated with acetaminophen (Figure 5d) are diverse; acute overdose treatment induced low urea production, vehicle albumin synthesis, increased aspartate aminotransferase (AST), and diminished alanine aminotransferase (ALT) and lactate dehydrogenase (LDH) in conventional two‐dimensional cultures. Similarly to conventional‐cultured hepatocytes, Exoliver‐cultured hepatocytes also exhibited decreased urea, ALT and LDH production; nevertheless a significant increase in albumin synthesis and a reduction in AST release to the culture media were observed in response to acetaminophen treatment.

Hepatocytes morphology after treatment with toxic drugs confirmed lack of viability in conventional two‐dimensional cultures (Figure 5e). Further mechanistic analysis of hepatocytes cell death in response to toxicants revealed high levels of necrosis, as suggested by elevated soluble keratin 18 (Figure 5f) in the culture media, with no differences in apoptosis‐related caspase‐cleaved keratin 18 (Figure 5g).

To further study the applicability of Exoliver assessing hepatotoxicity, the effects of 7‐day treatment with tolcapone were analyzed. As shown in Supporting Information Figure 4, and very similar to what was observed at Day 3, hepatocytes cultured by conventional methods exhibited a profound deregulation in viability and function as demonstrated by a marked decrease in urea and albumin synthesis, together with the increased release of transaminases and LDH. Interestingly, hepatocytes cultured in the device show higher resistance to this toxicant in comparison with conventional.

## 4 DISCUSSION

The current study demonstrates for the first time that it is possible to maintain primary human hepatocytes in vitro when cocultured with functional primary LSEC in a sinusoidal‐like milieu. The study has been developed using a liver‐resembling device that mimics the unique architecture of the liver sinusoid allowing layered coculture of multiple cell types with controlled endothelial shear stress stimulation and paracrine communications, as it occurs in the human liver.

We herein demonstrate that the benefits of this coculture system rely on the presence of functional LSEC. Indeed, the device benefits are mainly lost in both suboptimal Exoliver configurations: a perfused monoculture of hepatocytes or coculture of cells without biomechanical stimulation. In the first scenario, and although indirect flow stimulation per se might exert some beneficial effects on hepatocytes (Dash et al., 2013; Kang et al., 2015; Rashidi, Alhaque, Szkolnicka, Flint, & Hay, 2016), we observed that this configuration was inferior to the coculture of hepatocytes with flow‐stimulated LSEC. In the second situation, hepatocytes phenotype was lost in the absence of endothelial shear stress probably due to LSEC dedifferentiation upon isolation and in vitro culture (March, Hui, Underhill, Khetani, & Bhatia, 2009).

However, our investigations demonstrate that LSEC functional phenotype can be efficiently maintained under dynamic culture (Marrone et al., 2013; Shah et al., 1997), ultimately leading to hepatocytes maintenance (Dash et al., 2013). Underlying mechanisms of such protection may derive from the fact that LSEC cultured in static configurations become rapidly dysfunctional, driving molecular signaling to hepatocytes that ultimately promote, or at least do not prevent, their dedifferentiation. In addition, functional LSEC might release soluble factors or membrane‐embedded entities that contribute to maintain hepatocyte phenotype (Ding et al., 2010; Hu et al., 2014; Koch et al., 2017). In fact, upregulation of *hepcidin*/*hamp* in Exoliver cocultured hepatocytes (Supporting Information Figure 1D) supports angiocrine communication from functional LSEC. We cannot discard that future designs of the device, in which direct contact interactions between cells may be allowed as it occurs in the sinusoids, would give superior beneficial results than those herein described.

Interestingly, and most likely due to the singular design of the device, a relative gradient in oxygen along the culture area was observed (Supporting Information Figure 5A). Specialization of liver cells along the portal triad—central vein axis is known as zonation, and major drivers for such compartmentalization include nutrients, hormones, and growth factors, but specially oxygen. Because zonation directly affects macronutrient metabolism, morphology, and xenobiotic transformation in hepatocytes, oxygen gradient could indeed contribute to better reproduce the sinusoidal milieu and therefore to the maintenance of hepatocytes in the device (Kietzmann, 2017). Importantly, Exoliver‐cultured hepatocytes at the inflow area were enriched in genes predominantly expressed in periportal areas of the human liver, whereas hepatocytes at the outflow predominantly expressed pericentral typical genes (McEnerney et al., 2017; Figure 5b,c).

Considering the beneficial effects of this biosystem in preserving the phenotype of healthy human hepatocytes, we subsequently aimed at demonstrating its translational potential in two clinically relevant areas. Data demonstrating maintenance of the phenotype of human cirrhotic hepatocytes creates a new preclinical stage to test the efficacy of novel therapeutic options for the chronic liver disease. Indeed, the device may offer highly valuable information about the effects of a certain chemical entity in a human liver‐like environment just before administering it to humans. As an example, data from our team using the herein described device demonstrate that a caspase inhibitor that is currently at clinical evaluation for the treatment of chronic liver disease improves human cirrhotic hepatocytes without evidence of toxicity (Gracia‐Sancho, Contreras, Vila, Garcia‐Caldero, Spada, & Bosch, 2016).

Further translational studies focused on the field of drug‐induced liver injury. Interestingly, Exoliver‐cultured hepatocytes responded significantly different to hepatotoxic drugs than dedifferentiated cells. These data suggest that concentrations of drugs previously proposed to be hepatotoxic in vitro may not truly promote cell death when tested in functional hepatocytes. Vice versa, it is now conceivable that some drugs that were withdrawn due to toxicity in two‐dimensional primary cultures could have not been harmful if tested in a more physiological environment. Although primary hepatocytes are considered the current gold standard for short‐term in vitro toxicant testing, they are severely hindered by the lack of three‐dimensional organization, nonparenchymal cells, nutrient access, and cell–cell interactions, which can be found in liver‐on‐a‐chip devices. For this reason, preclinical research should consider the analysis of toxicity in physiologically resembling devices, which may provide valuable data that would complement results obtained in two‐dimensional hepatocytes cultures.

We are aware that our study has limitations; probably the most important is that our device does not entirely recapitulate the diversity of cells found in the liver sinusoid. Adding extra cell layers, with hepatic stellate cells and/or macrophages, would probably increase its biological resemblance. Nevertheless, our data show that adding perfused LSEC per se is able to maintain hepatocytes phenotype, suggesting that LSEC play a major role in hepatocyte homeostasis.

It is true that future perspectives on liver bioengineering research are set in generating improved in vitro culture systems for modeling human diseases and performing valuable assays. The development of in vitro devices that address systemic human biology using liver‐resembling devices in combination with other organ biosystems is highly needed (Coppeta et al., 2017; Maschmeyer et al., 2015). The herein described platform may contribute to create these body‐on‐a‐chip structures that will ultimately allow a global understanding of prodrugs and metabolites’ effects in various organs.

To sum up, our study describes a novel bioengineered device that resembles the human liver in vitro, currently representing the preclinical setup closest to the bedside. Altogether encourages its applicability for the study of liver diseases and toxicology.

## CONFLICTS OF INTEREST

The authors of this manuscript have no conflict of interests to disclose as described by Biotechnology & Bioengineering.

## AUTHORS CONTRIBUTION

M. O.‐R. conceived the study, designed the research, performed the experiments, analyzed the data, and wrote the manuscript. A. F.‐I., A. M., and R. M.‐D. performed experiments. X. I. prepared three‐dimensional figures, manufactured membranes, and critically revised the manuscript. V. M. and C. F. procured human liver tissue for the study. C. P. and J. B. critically revised the manuscript. R. V. conceived ideas, critically revised the manuscript, and obtained funding. J. G.‐S. conceived the study, designed and directed the research, wrote the manuscript, and obtained funding. All authors edited and reviewed the final manuscript.

## Supporting information

Supporting information

Supporting information

Supporting information

Supporting information

Supporting information

Supporting information

Supporting information
