# Supplementary figures and images for: Resemblance of the human liver sinusoid in a fluidic device with biomedical and pharmaceutical applications
Source: Biotechnol Bioeng. 2018 Jul 13;115(10):2585–94. doi: 10.1002/bit.26776 (PMC6220781; doi:10.1002/bit.26776)

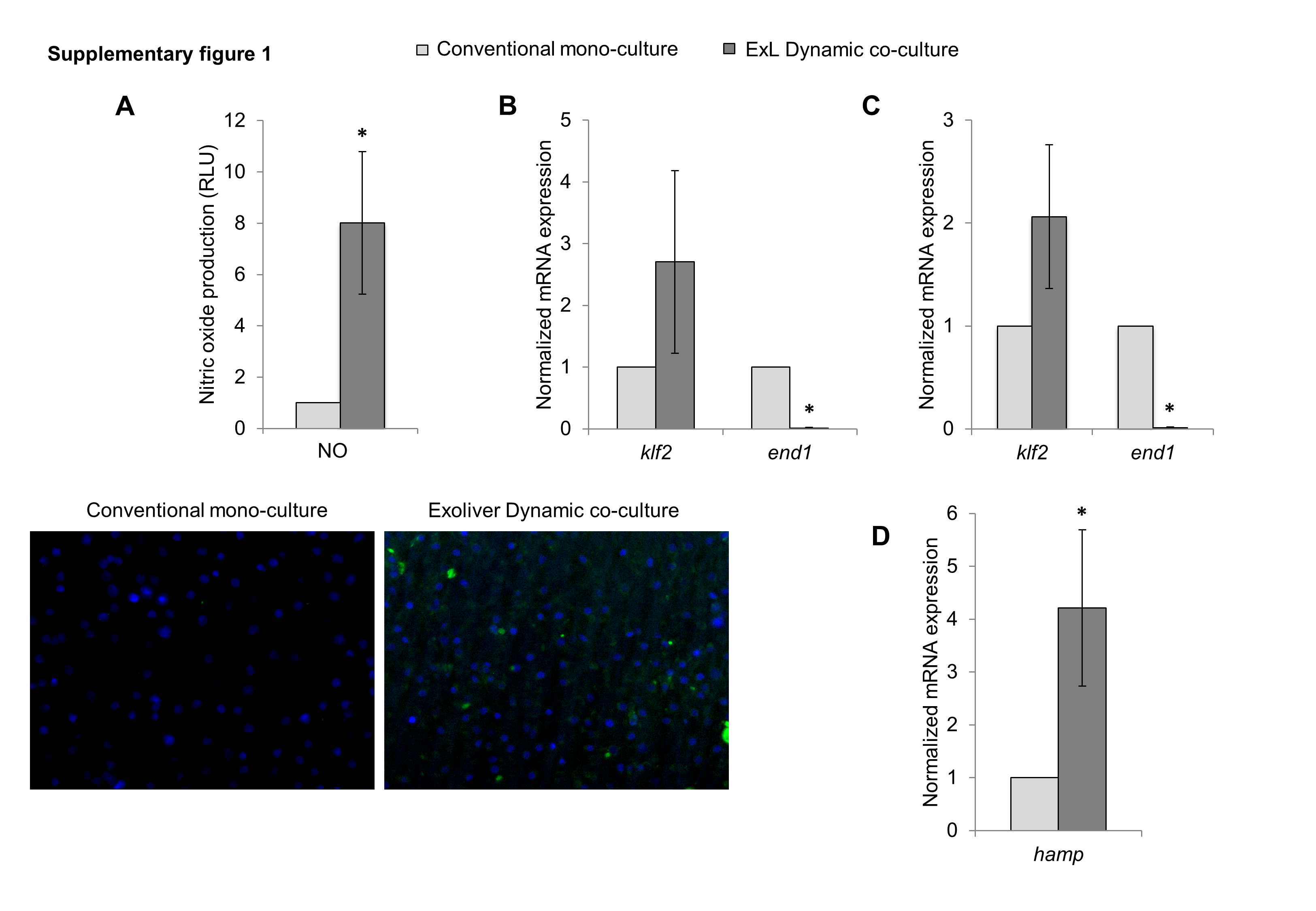

Supplement: Supplementary file 1 — Supporting information [file BIT-115-2585-s001.TIF]

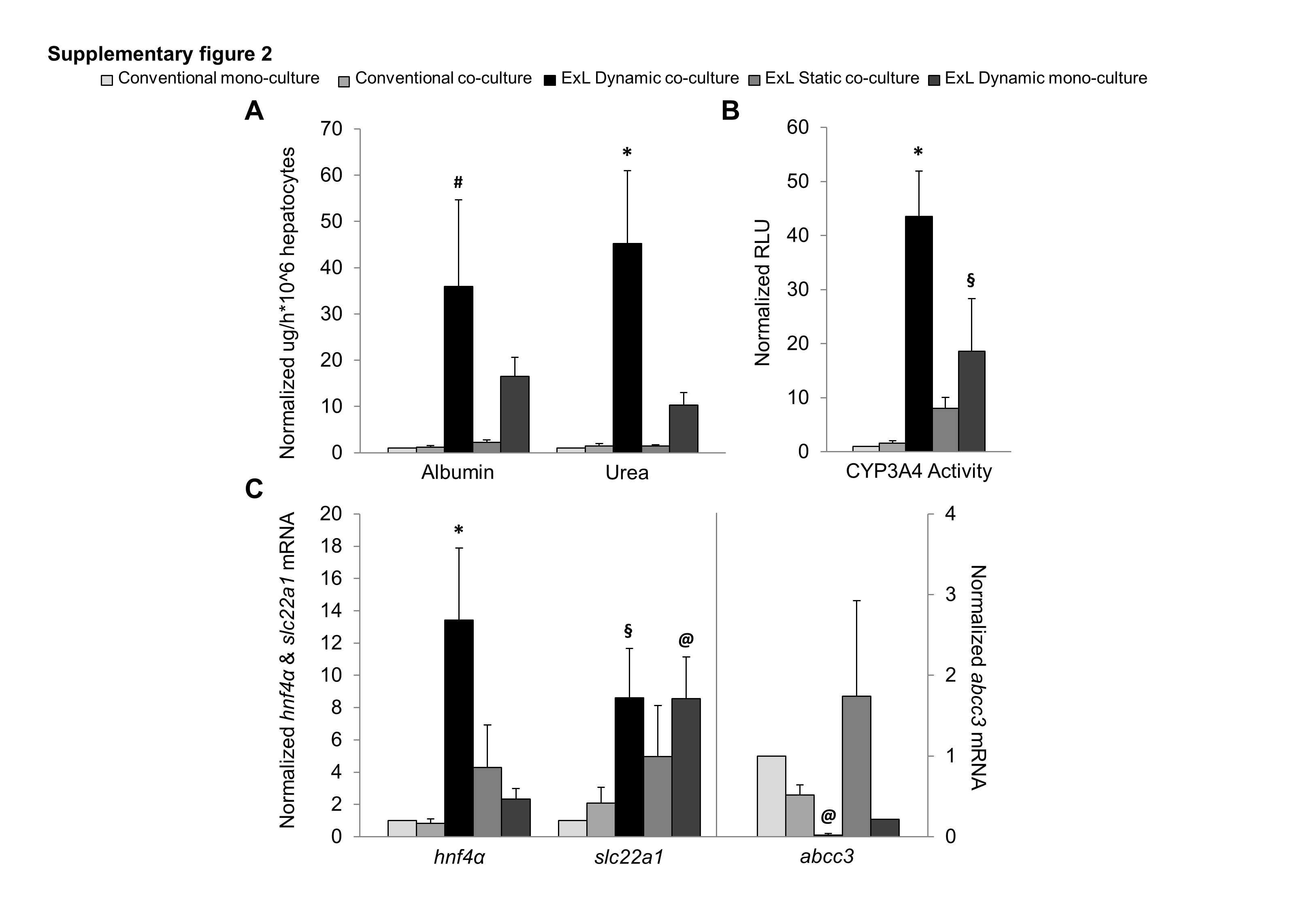

Supplement: Supplementary file 2 — Supporting information [file BIT-115-2585-s002.TIF]

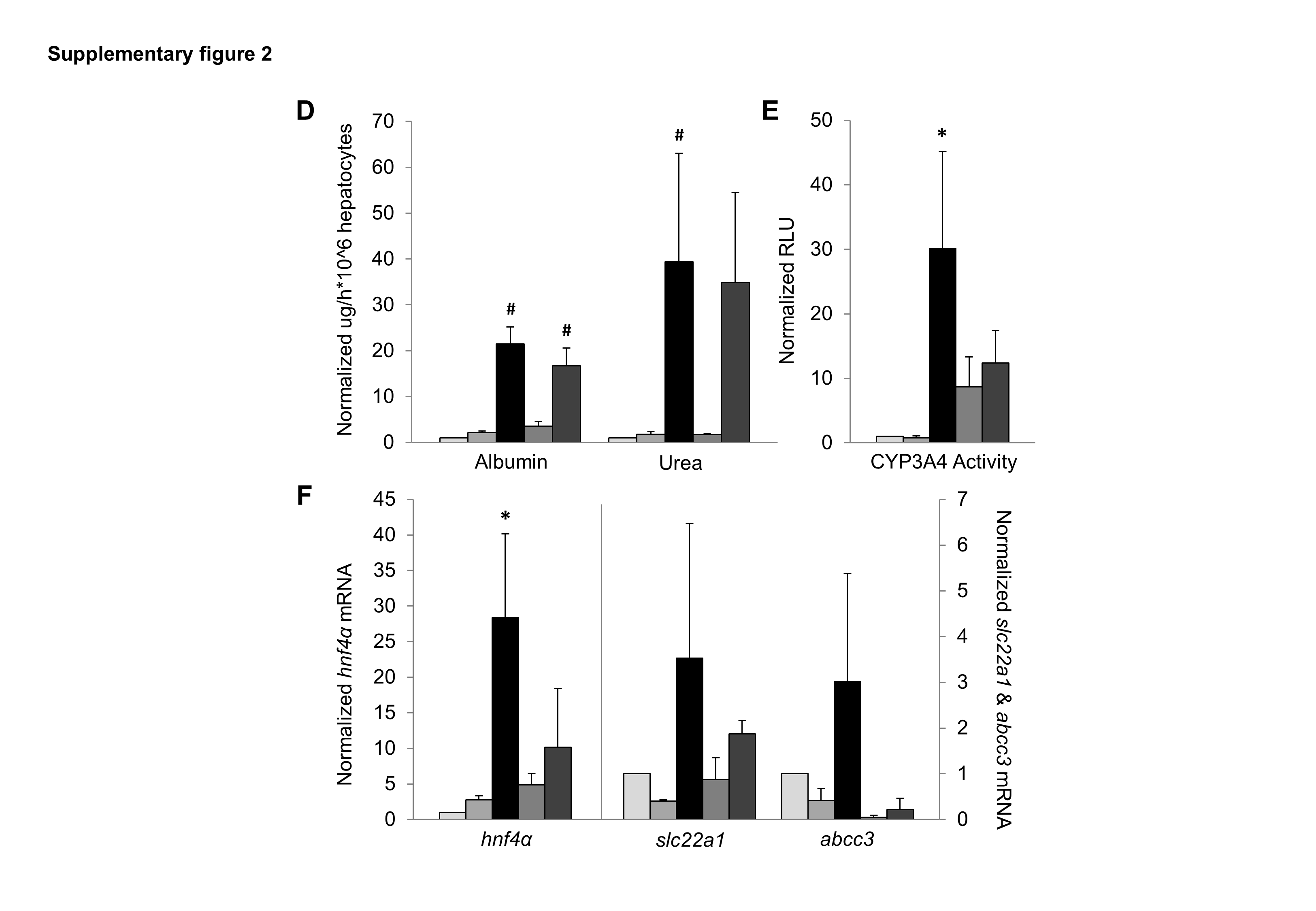

Supplement: Supplementary file 3 — Supporting information [file BIT-115-2585-s003.TIF]

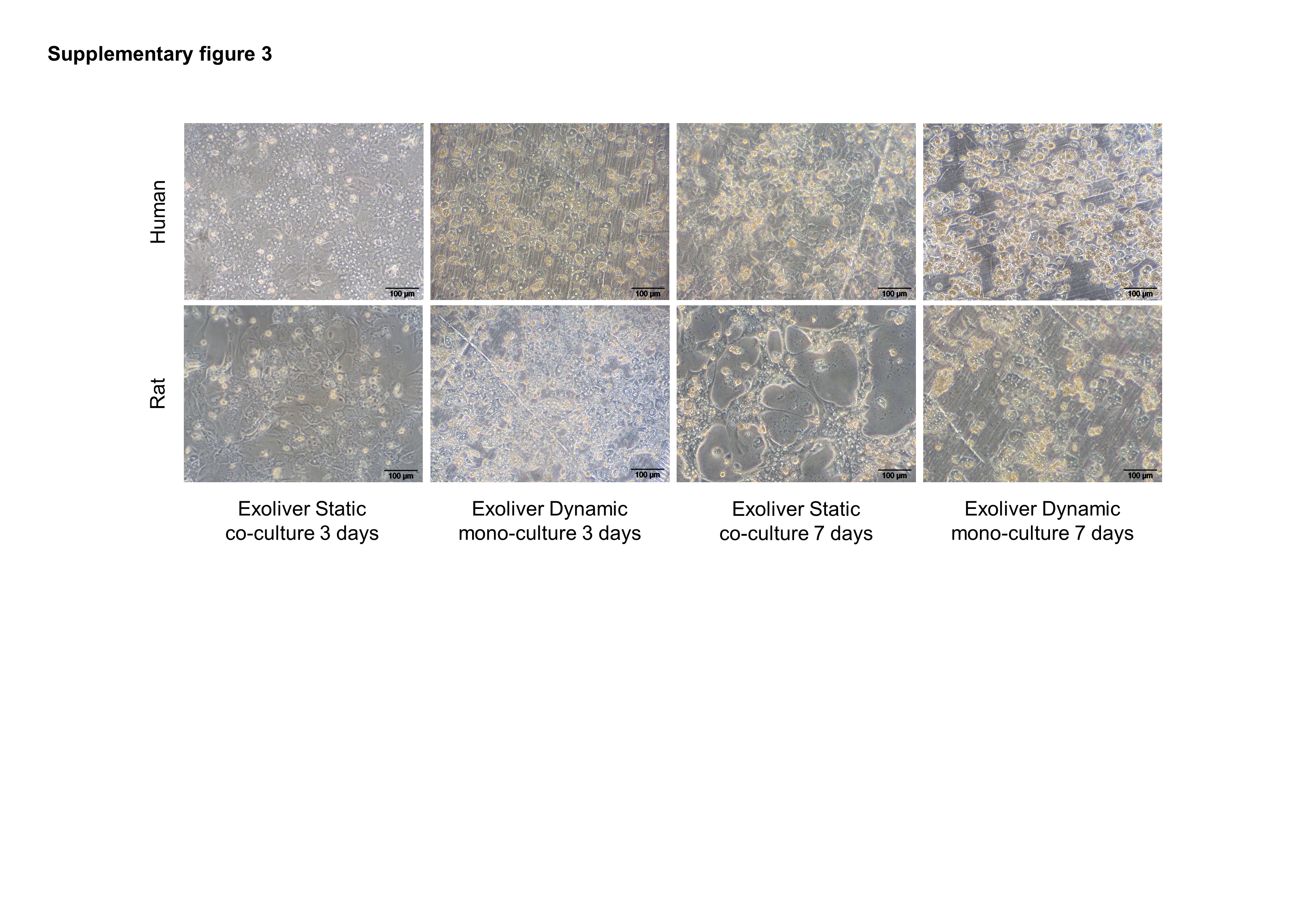

Supplement: Supplementary file 4 — Supporting information [file BIT-115-2585-s004.TIF]

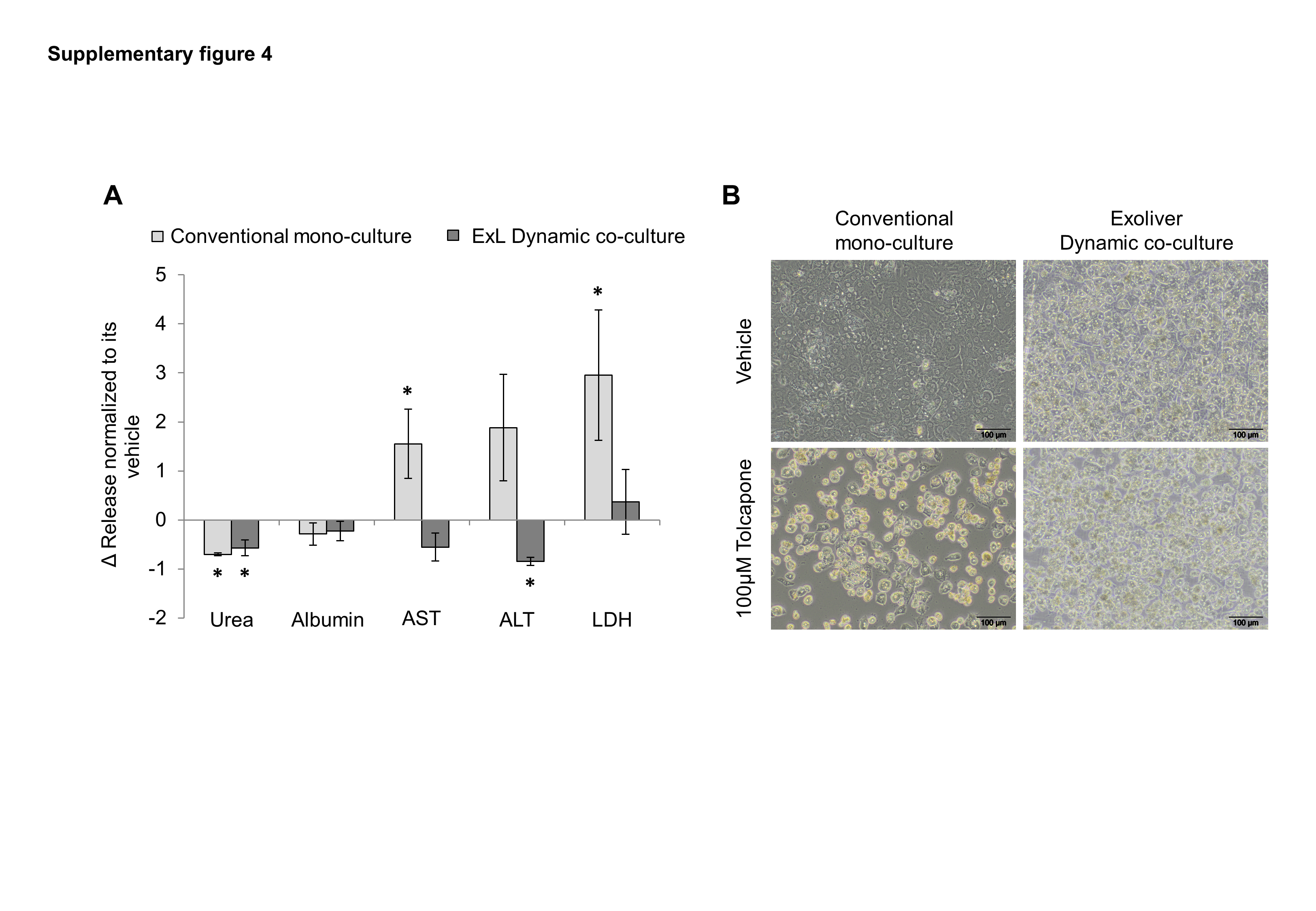

Supplement: Supplementary file 5 — Supporting information [file BIT-115-2585-s005.TIF]

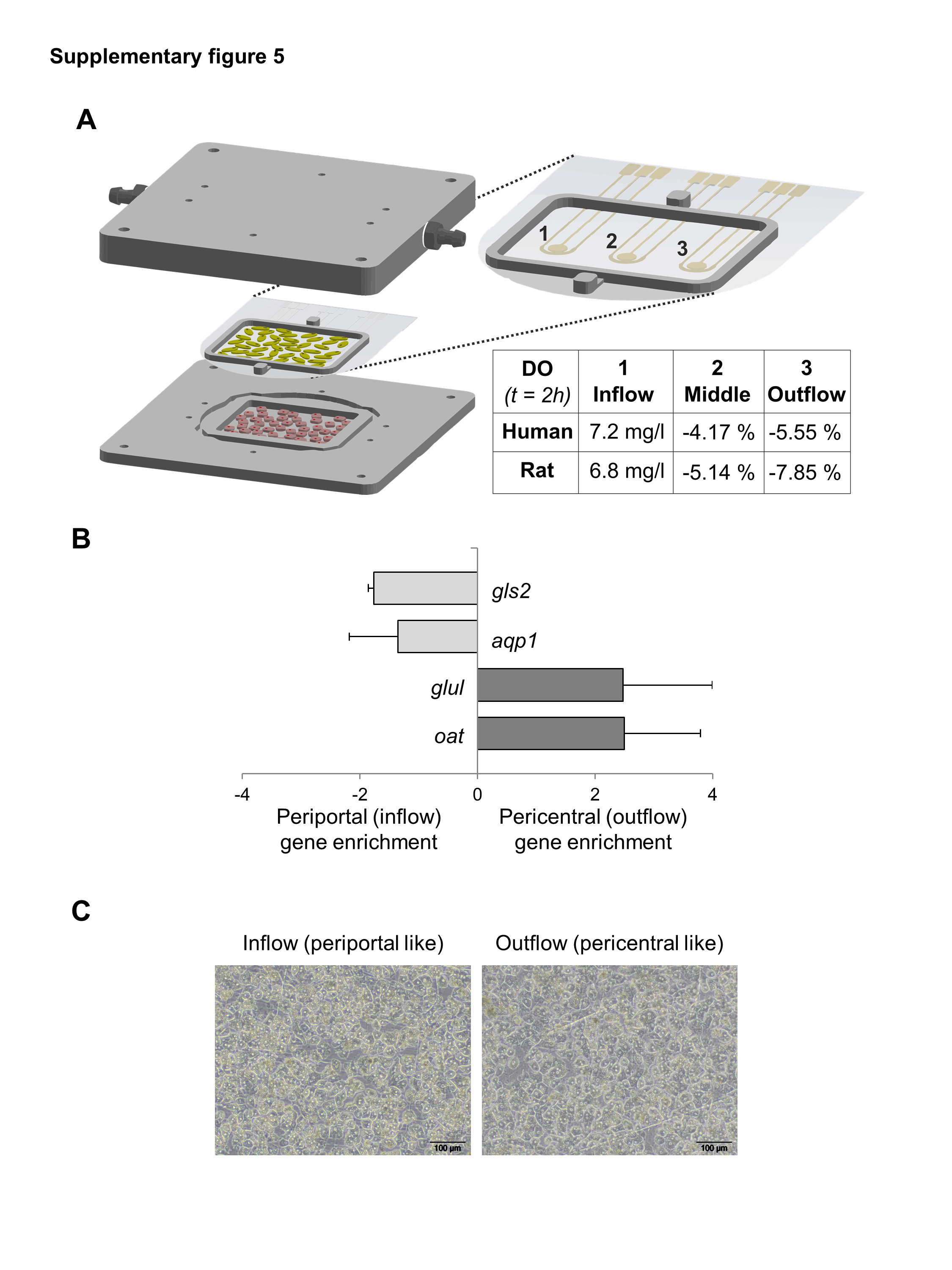

Supplement: Supplementary file 6 — Supporting information [file BIT-115-2585-s006.TIF]
