# Supplementary material for: Resemblance of the human liver sinusoid in a fluidic device with biomedical and pharmaceutical applications
Source: Biotechnol Bioeng. 2018 Jul 13;115(10):2585–94. doi: 10.1002/bit.26776 (PMC6220781; doi:10.1002/bit.26776)
Supplement: Supplementary file 7 — Supporting information [file BIT-115-2585-s007.docx]

**Resemblance of the human liver sinusoid in a fluidic device with biomedical and pharmaceutical applications**

Martí Ortega-Ribera, Anabel Fernández-Iglesias, Xavi Illa, Ana Moya, Víctor Molina, Raquel-Maeso Díaz, Constantino Fondevila, Carmen Peralta, Jaume Bosch, Rosa Villa, Jordi Gracia-Sancho

**Supplementary methods**

**Tissue digestion**

Livers were perfused through major vessels (human tissue) or the portal vein (rats) for 10 min at a flow rate of 20 mL/min at 37 °C with Hanks without Ca^+2^ and Mg^+2^ containing 12 mM hepes (H3375, Sigma) pH 7.4, 0.6 mM ethylene glycol-bis (2-aminoethylether)-N,N,N′,N′-tetraacetic acid (E4378, Sigma) and 0.23 mM bovine serum albumin (BSA; A1391,0100, Applichem). Then, perfused for 30 min at a flow rate of 5-20 mL/min at 37°C with 0.015% collagenase A (103586, Roche) Hanks containing 12 mM hepes (pH 7.4) and 4 mM CaCl_2_. The resultant digested liver was excised and *in vitro* digestion was performed at 37°C with 0.01% collagenase A, Hank’s containing 12 mM hepes (pH 7.4) and 4 mM CaCl_2_ for 10 min. Disaggregated tissue was filtered using 100 µm nylon strainer, collected in cold Krebs buffer and centrifuged at 50 g for 5 min. Hepatocytes were contained in the pellet while non-parenchymal cells were found in the supernatant. Part of the healthy hepatic cells isolation was performed using disaggregated multicellular preparations from ReadyCell & Cytes Biotechnology.

**Isolation of primary rat and human hepatocytes**

Pellet containing hepatocytes was rinsed three times with Hanks Balanced Solution salt (HBSS; H8264, Sigma) or HBSS with 0,5% BSA for human hepatocytes following 50 g centrifugation for 5 min. Hepatocytes above 80% viability and 95% purity, were cultured in Dulbecco’s Modified Eagle’s Medium (DMEMF12; 11320074, Gibco) supplemented with 10% Fetal Bovine Serum (FBS; 04-001-1A, Reactiva), 1% penicillin plus 1% streptomycin (03-331-1C, Reactiva), 2 mM L-glutamine (25030-024, Gibco), 1% amphotericin B (03-029-1C, Reactiva), 1 µM dexamethasone (D4902, Sigma) and 1 µM insulin (103755, HCB) and were plated in 0.1 mg/ml collagen type 1 rat tail (A10483-01, GIBCO) coated substrates (plasma treated Poly methyl methacrylate plates for dynamic cultures and 35 mm petri dish for conventional cultures) at a density of 109.000 cells/cm^2^ and maintained at 37°C in a humidified atmosphere of 5% CO_2_ for 4h. After 4h, cells were rinsed twice with Dulbecco’s phosphate-buffered saline (DPBS; 02-023-1A, Reactiva) and medium was changed to 1 nM dexamethasone and 2% FBS with same composition of the other supplements and were maintained overnight (O/N) at the previous described conditions.

**Isolation of primary rat LSEC**

The supernatant containing non-parenchymal cell fraction was centrifuged at 800 g for 10 min at 4°C and resuspended in DPBS and centrifuged at 800 g for 25 min through a two-step 25-50% Percoll gradient (17-0891-02, GE Healthcare) at 4°C. The interface of the gradient was enriched in Kupffer cells and LSEC. This cell fraction was diluted in DPBS and centrifuged at 800g for 10 min. The cell pellet was resuspended in Roswell Park Memorial Institute medium (RPMI 1640 without L-glutamine; 01-100-1A, Reactiva) supplemented with 10% FBS, 1% penicillin plus 1% streptomycin, 2 mM L-glutamine, 1% amphotericin B, 1% endothelial cell growth supplement (ECGS; BT-203, Biomedical Technologies) and 100 mg/ml heparin (H3393, Sigma), seeded in plastic dishes and incubated for 30 min at 37°C in humid atmosphere with 5% CO_2_ in order to enhance LSEC purity by selective adherence of Kupffer cells. Non-adhered cells were seeded in collagen-coated substrates (Polytetrafluoroethylene membranes for dynamic cultures or 6-well transwell for conventional co-cultures) and maintained 45 min at the previous incubation conditions. Afterwards, cells were washed twice with DPBS and left O/N (37°C, 5% CO_2_) in RPMI-1640.

LSEC exhibited 93% purity (positive for Reca-1 and presence of fenestrae) and viability above 95%. LSEC differentiated phenotype was assessed in terms of real time nitric oxide (NO) bioavailability using DAF-FM (D23844, Invitrogen) staining (Gracia-Sancho et al., 2007).

**Isolation of primary human LSEC**

The supernatant containing non-parenchymal cell fraction was centrifuged at 800 g for 10 min at 4°C. The obtained pellet was resuspended in 10 ml DPBS and incubated with 10 µL human CD32 primary antibody (10R-CD32eHU, Fitzgerald) for 30 min for subsequent immunomagnetic separation of LSEC with Dynabeads® CELLection Pan Mouse IgG Kit (11531D, Thermo Scientific, Oslo, Norway) following manufacturer’s instructions. LSEC were resuspended in RPMI-1640 supplemented with 10% FBS, 1% penicillin plus 1% streptomycin, 2 mM L-glutamine, 1% amphotericin B, 1% ECGS and 100 mg/ml heparin and seeded in collagen-coated substrates (Polytetrafluoroethylene membranes for dynamic cultures or 6-well transwell for conventional co-cultures) and left for 1 h (37°C, 5% CO_2_) in RPMI-1640 to enable live cells attachment (Oie, Snapkov, Elvevold, Sveinbjornsson, & Smedsrod, 2016). LSEC differentiated phenotype was determined analyzing the expression of the endothelial transcription factor krüppel-like factor 2 (*klf2)* and the vasoconstrictor endothelin-1 (*end1*) by qPCR as described below.

**Hepatotoxicity assay**

Acute drug–induced toxicity experiments were performed in healthy human hepatocytes with 4 hepatotoxic drugs at the following concentrations: 100 µM for troglitazone (T2573) and tolcapone (SML0150), 1 mM for diclofenac (D6899) and 40 mM for acetaminophen (A7085) all commercially provided by Sigma-Aldrich. Concentrations were based on previous bibliography using conventional mono-culture of hepatocytes (Allen, Khetani, & Bhatia, 2005; Khetani & Bhatia, 2008; Knöspel et al., 2016). Drug addition to the culture media was maintained along 24h from day 2 to day 3 post-isolation. Hepatotoxic effect of the drug in conventional mono-culture was compared to Exoliver dynamic co-culture configuration and assessed as AST, ALT, LDH release to the culture media and albumin and urea production. Validation experiments at 7 days of culture were carried out using 100 µM tolcapone.

**RNA isolation, reverse transcription and RTqPCR**

Total RNA from hepatocytes or LSEC was extracted and preserved at -80°C with RLT solution (Qiagen) containing 10 mM β-mercaptoethanol until isolation. Total RNA was isolated and purified using RNeasy Mini Kit (74104, Qiagen) according to manufacturer’s instructions. RNA was quantified using Nanodrop software (ND1000, ThermoScientific) and reverse transcribed to cDNA using Taqman Fast Universal PCR Master Mix (4352042, Applied Biosystems) previous elimination of genomic DNA of the sample. cDNA templates were amplified by real-time TaqMan polymerase chain reaction on an ABI Prism 7900HT Fast Detection System (Applied Biosystems).

Expression of *hnf4α* (Rn04339144_m1 and Hs00230853_m1), *abcc3* (Rn01452854_m1 and Hs00978473_m1), *slc22a1* (Rn00562250_m1 and Hs00427552_m1), *klf2* (Rn01420495_g1 and Hs00360439_g1), *end1* (Rn00561129_m1 and Hs00174961_m1), *hamp* (Rn00584987_m1), *glul* (Hs00365928_g1), *oat* (Hs00236852_m1), *gls2* (Hs00998733_m1) and *aqp1* (Hs01028916_m1) were analyzed using predesigned gene expression assays from Applied Biosystems (Thermo Fisher Scientific) and reported relative to endogenous controls *gapdh* (Rn01775763_g1) for rat or *18S* (Hs99999901_s1) for human samples. All PCR reactions were performed in duplicate and using nuclease-free water as controls.

**Cell supernatant markers**

Culture media from all experimental conditions after 3 or 7 days of culture were sampled. Albumin, blood urea nitrogen (BUN), transaminase AST and ALT and lactate dehydrogenase (LDH) were measured using standard methods at the Hospital Clínic of Barcelona’s CORE laboratory. BUN values were converted to urea as 2.1428 mg/dl BUN =1 mg/dl urea.

**Cell death analyses**

Global cell death (necrosis + apoptosis) was evaluated as release of soluble keratin 18 in cell supernatants using the M65^®^ ELISA (10020, Peviva). M30 Apoptosense^®^ ELISA (10011, Peviva) was used to determine the apoptosis-associated caspase-cleaved keratin 18 levels in supernatants (Ku, Strnad, Bantel, & Omary, 2016).

**Cytochrome P450 family 3 subfamily A member 4 (CYP3A4) activity**

Phase I detoxification capacity of hepatocytes was analyzed using P450-Glo™ CYP3A4 Assay following manufacturer’s instructions (V8901, Promega). Briefly, hepatocytes after 3 or 7 days of culture in conventional methods or Exoliver were rinsed twice with DPBS and incubated with culture media containing 50 µM Luciferin-PFBE at 37°C for 4h. Then supernatant was collected and neutralized with Luciferin Detection Reagent vol (1/1). After incubation for 30 min at RT, plate luminescence was read in a luminometer (Orion II Microplate Luminometer, Germany). Samples luminescence were corrected subtracting background luminescence.

**Oxygen zonation**

Dissolved oxygen (DO) gradient within the device was assessed using manufactured oxygen sensors described in (Moya et al., 2016). Simultaneous *in situ* measurements above the inflow, middle and outflow culture areas were performed after 2 hours of stabilization of the culture (**Supplementary figure 5**). Inkjet printing was the selected manufacturing method for the integration of the DO sensors on the cell culture membrane. DO sensor operation was based on amperometric measurements consisting on an electrochemical three-electrode system (reference, auxiliary and working electrodes). In this type of measurements the sensor is polarized at a potential in which oxygen is reduced, and this reduction current generated at the working electrode is directly proportional to the oxygen content in the tested solution. Sensors were manufactured in a micrometric range (working electrode diameter of 300 µm) blocking just a small area of the culture membrane. A commercial multichannel potentiostat (1030A, CH Instruments, USA) was used for simultaneous DO measurements.

**Supplementary results**

**Exoliver maintains rat hepatocyte phenotype and function**

Maintenance of rat hepatocytes phenotype was assessed in 5 experimental conditions (**Figure 1 top**): hepatocytes cultured in 2 conventional configurations (mono-culture and co-culture with LSEC) and cultured within Exoliver in 3 different configurations: co-culture with LSEC stimulated with continuous and homogenous shear stress (optimal condition), co-culture without shear stress (which leads to LSEC dysfunction **Supplementary figure 1**) and hepatocytes mono-culture with indirect flow stimulus (without paracrine interactions from LSEC).

Synthetic capacity of hepatocytes was evaluated as active albumin and urea production and release to the culture media. Hepatocytes cultured in the Exoliver dynamic co-culture condition showed higher albumin and urea production when compared to all static conditions, both after 3 days (**Supplementary figure** **2A**) or 7 days (**Supplementary figure** **2D**) of culture. When comparing both dynamic configurations; optimal Exoliver configuration showed superior urea and albumin synthetic capacity than dynamic mono-culture configuration.

Hepatocytes cultured under dynamic co-culture configuration using Exoliver exhibited higher Phase I enzymatic activity as shown by increased CYP3A4 activity compared to all other conditions after 3 days (**Supplementary figure** **2B**) or 7 days (**Supplementary figure** **2E**) of culture. Although CYP3A4 activity in the dynamic mono-culture configuration was partially increased after 3 days of culture, this was no longer observed after 7 days of culture, therefore reinforcing the concept of hepatocytes maintenance through paracrine interactions from functional LSEC in the dynamic co-culture condition.

Hepatocytes phenotype was assessed by means of expression of the master regulator *hnf4α*, and the transporters *abcc3* and *slc22a1*. Optimal Exoliver configuration (dynamic co-culture) delayed loss of expression of the transcription factor *hnf4α* after 3 (**Supplementary figure** **2C**) and 7 days (**Supplementary figure** **2F**) of culture when compared to all other analyzed conditions. Exoliver dynamic mono-culture configuration was not able to significantly maintain *hnf4α* expression along days of culture. Finally, characteristic loss of *slc22a1* and increase in *abcc3* mRNA expression during *in vitro* hepatocytes dedifferentiation were prevented after 3 days of culture inside Exoliver compared to conventional *in vitro* cultures. At 7 days of culture, there was a trend towards preventing loss of *slc22a1* transporter expression in Exoliver optimal condition although *abcc3* increase was not delayed (**Supplementary figure** **2F**). The beneficial effects of co-culturing hepatocytes with shear stress-stimulated LSEC using Exoliver were not seen in the absence of flow stimulation of the endothelial layer.

Conventional mono- and co-culture configurations showed no significant differences in any of the studied parameters neither after 3 days or 7 days of culture.

**Reference list**

Allen, J. W., Khetani, S. R., & Bhatia, S. N. (2005). In vitro zonation and toxicity in a hepatocyte bioreactor. *Toxicological Sciences*, *84*(1), 110–119. http://doi.org/10.1093/toxsci/kfi052

Gracia-Sancho, J., Laviña, B., Rodríguez-Vilarrupla, A., García-Calderó, H., Bosch, J., & García-Pagán, J. C. (2007). Enhanced vasoconstrictor prostanoid production by sinusoidal endothelial cells increases portal perfusion pressure in cirrhotic rat livers. *Journal of Hepatology*, *47*(2), 220–227. http://doi.org/10.1016/j.jhep.2007.03.014

Khetani, S. R., & Bhatia, S. N. (2008). Microscale culture of human liver cells for drug development. *Nature Biotechnology*, *26*(1), 120–6. http://doi.org/10.1038/nbt1361

Knöspel, F., Jacobs, F., Freyer, N., Damm, G., De Bondt, A., van den Wyngaert, I., … Zeilinger, K. (2016). In Vitro Model for Hepatotoxicity Studies Based on Primary Human Hepatocyte Cultivation in a Perfused 3D Bioreactor System. *International Journal of Molecular Sciences*, *17*(4), 584. http://doi.org/10.3390/ijms17040584

Ku, N. O., Strnad, P., Bantel, H., & Omary, M. B. (2016). Keratins: Biomarkers and modulators of apoptotic and necrotic cell death in the liver. *Hepatology*, *64*(3), 966–976. http://doi.org/10.1002/hep.28493

Moya, A., Sowade, E., del Campo, F. J., Mitra, K. Y., Ramon, E., Villa, R., … Gabriel, G. (2016). All-inkjet-printed dissolved oxygen sensors on flexible plastic substrates. *Organic Electronics: Physics, Materials, Applications*, *39*, 168–176. http://doi.org/10.1016/j.orgel.2016.10.002

Oie, C. I., Snapkov, I., Elvevold, K., Sveinbjornsson, B., & Smedsrod, B. (2016). FITC Conjugation Markedly Enhances Hepatic Clearance of N-Formyl Peptides. *PloS One*, *11*(8), e0160602. http://doi.org/10.1371/journal.pone.0160602
